# Clinical response after two cycles compared to HER2, Ki-67, p53, and bcl-2 in independently predicting a pathological complete response after preoperative chemotherapy in patients with operable carcinoma of the breast

**DOI:** 10.1186/bcr1989

**Published:** 2008-04-01

**Authors:** Gunter von Minckwitz, Hans-Peter Sinn, Günter Raab, Sibylle Loibl, Jens-Uwe Blohmer, Holger Eidtmann, Jörn Hilfrich, Elisabeth Merkle, Christian Jackisch, Serban D Costa, Angelika Caputo, Manfred Kaufmann, the German Breast Group (GBG)

**Affiliations:** 1 Dept. of Gynecology and Obstetrics, Goethe University, Frankfurt am Main, Germany; 2 German Breast Group, Neu-Isenburg, Germany; 3 Dept. of Pathology, University of Heidelberg, Germany; 4 Practicing Gynecologist, Maximilinastrasse, Munich, Germany; 5 Dept. of Gynecology St. Getrauden Hospital, Berlin, Germany; 6 Dept. of Gynecology, University of Kiel, Germany; 7 Henrietten-Stiftung Gynecological Hospital, Hannover, Germany; 8 Am Berg Gynecological Hospital, Stuttgart, Germany; 9 Dept. of Gynecology, Stadtkrankenhaus Offenbach, Germany; 10 Dept. of Gynecology and Obstetrics, University of Magdeburg Germany; 11 Dept. of Medical Biometry and Statistics, University Medical Center, Freiburg, Germany

## Abstract

**Introduction:**

To investigate the predictive value of clinical and biological markers for a pathological complete remission after a preoperative dose-dense regimen of doxorubicin and docetaxel, with or without tamoxifen, in primary operable breast cancer.

**Methods:**

Patients with a histologically confirmed diagnosis of previously untreated, operable, and measurable primary breast cancer (tumour (T), nodes (N) and metastases (M) score: T2-3(≥ 3 cm) N0-2 M0) were treated in a prospectively randomised trial with four cycles of dose-dense (bi-weekly) doxorubicin and docetaxel (ddAT) chemotherapy, with or without tamoxifen, prior to surgery. Clinical and pathological parameters (menopausal status, clinical tumour size and nodal status, grade, and clinical response after two cycles) and a panel of biomarkers (oestrogen and progesterone receptors, Ki-67, human epidermal growth factor receptor 2 (HER2), p53, bcl-2, all detected by immunohistochemistry) were correlated with the detection of a pathological complete response (pCR).

**Results:**

A pCR was observed in 9.7% in 248 patients randomised in the study and in 8.6% in the subset of 196 patients with available tumour tissue. Clinically negative axillary lymph nodes, poor tumour differentiation, negative oestrogen receptor status, negative progesterone receptor status, and loss of bcl-2 were significantly predictive for a pCR in a univariate logistic regression model, whereas in a multivariate analysis only the clinical nodal status and hormonal receptor status provided significantly independent information. Backward stepwise logistic regression revealed a response after two cycles, with hormone receptor status and lymph-node status as significant predictors. Patients with a low percentage of cells stained positive for Ki-67 showed a better response when treated with tamoxifen, whereas patients with a high percentage of Ki-67 positive cells did not have an additional benefit when treated with tamoxifen. Tumours overexpressing HER2 showed a similar response to that in HER2-negative patients when treated without tamoxifen, but when HER2-positive tumours were treated with tamoxifen, no pCR was observed.

**Conclusion:**

Reliable prediction of a pathological complete response after preoperative chemotherapy is not possible with clinical and biological factors routinely determined before start of treatment. The response after two cycles of chemotherapy is a strong but dependent predictor. The only independent factor in this subset of patients was bcl-2.

**Trial registration number:**

NCT00543829

## Introduction

Preoperative (neo-adjuvant) chemotherapy in primary operable breast cancer has been shown to produce an outcome equivalent to that of postoperative (adjuvant) chemotherapy [1,2]. In case of neo-adjuvant therapy, patients with a complete remission of the primary tumour have a better prognosis than patients with a partial remission, or those with stable or progressive disease [3]. This has led to the hypothesis that the response of the primary tumour in the breast parallels the response of distant micrometastases, and that it can therefore be used as a surrogate parameter for clinical outcome. Randomised preoperative trials have, in contrast to trials in metastatic disease, the advantage of offering a relatively homogeneous population of patients without previous treatment; hence, the therapeutic effect can be evaluated more precisely and at an early stage due to the pathological examination that takes place at the time of surgery. This setting can be described as an *in vivo *chemosensitivity test. *In vitro *studies have identified a large number of determinants that are involved in mechanisms of resistance or sensitivity to chemotherapy [4]. The impact of these parameters has not been established for routine use for the effect of chemotherapy in humans. Preoperative treatment of breast cancer is ideal for the evaluation of the predictive value of these molecular markers, as tumour tissue can be obtained before and after treatment [5].

The oestrogen and progesterone receptor content of breast carcinomas have been regarded as the only established molecular markers capable of predicting the response to endocrine treatment in large-scale trials [6,7]. In preoperative trials, negative estrogens receptor status is strongly correlated to an increased sensitivity of a preoperative chemotherapy [8]. The mechanisms underlying these effects are not fully understood but *in vitro *studies have shown that ER signalling can increase levels of bcl-2 and induce anthracycline resistance [9].

Ki-67 is a nuclear antigen expressed in G_1_, S and G_2 _phase but not in G_0 _or the resting phase of the cell cycle [10]. It has become established as a proliferation marker in breast cancer. A high level of proliferation activity has been found to have predictive value for the response to preoperative chemotherapy [8]. Changes in the relative proportion of Ki-67 positive cells have been observed early after preoperative chemotherapy. A significant decrease 14 days after the initiation of neo-adjuvant treatment with tamoxifen correlated with a better chance of tumour response [11,12].

The prognostic and predictive value of human epidermal growth factor receptor 2 (HER2) has been investigated in a large number of publications [13] and was considered as a marker of resistance for example, for the cyclophosphamide, methotrexate, 5-fluorouracil (CMF) regimen or tamoxifen. Only recently, overexpression of HER2 was correlated with a higher sensitivity to taxanes [14].

Non-functional mutated p53 accumulates in the nucleus of tumour cells, where it can be detected by immunohistochemistry. However, the antigenic site of the protein is truncated in 40% of the cases and cannot be detected by the antibody used for immunohistochemistry. Undifferentiated tumours that are negative for p53 have a high possibility for an allelic loss or nonsense mutations. However, findings regarding the predictive value of p53 mutations in breast cancer have been contradictory. A large adjuvant trial of 595 patients showed that p53 mutations were predictive for a better effect of a higher dose of doxorubicin [15], but this was not confirmed in a preoperative trial of 329 patients [16].

The oncogene Bcl-2 is classically associated with follicular lymphomas and appears to be associated with chemoresistance in these types of tumours [17]. It is a cytosolic expressed protein that interacts with p53 and other proteins and act as inhibitor of apoptosis. Bcl-2 overexpression is described in approximately 80% of primary breast cancer [18] but a clear association with a chemoresistance has not been shown to date. Therefore, the situation in breast cancer is less clear-cut and needs further investigation [19].

Based on the promising results of a pilot phase IIa trial investigating a preoperative dose-dense combination regimen of doxorubicin and docetaxel (dose-dense Adriblastin and Taxotere, ddAT) in primary breast tumours ≥ 3 cm in diameter [20], the German Preoperative Adriamycin-Docetaxel (GEPARDO) group has initiated a prospective, randomised, controlled multi-centre trial for further evaluation of the safety of this dose-dense schedule, with or without simultaneous administration of tamoxifen [21]. The aim of the trial was to increase the pCR rate by simultaneously adding tamoxifen to the chemotherapy. The results in relation to compliance, toxicity, and efficacy have been reported previously.

The initially defined aim of the study was to assess various biomarkers: oestrogen receptor (ER) and progesterone receptor (PgR) content, Ki-67, HER2, p53 and bcl-2 in tissue obtained from core-cut biopsies before start of treatment, and to assess the value of these for predicting a pathological complete response in comparison with various clinical and pathological parameters.

## Methods

### Patient population

All patients had to meet the following major inclusion criteria to be enrolled in the clinical trial: unilateral primary carcinoma of the breast, confirmed histologically by core-cut needle or incisional biopsy (fine-needle aspiration was not considered sufficient); a tumour measurable two-dimensionally by mammography, breast ultrasound or breast MRI; a primary tumour ≥ 3 cm in its largest diameter (in patients with multifocal or multicentric breast cancer, the largest lesion was measured); no evidence of distant metastases (as confirmed by chest radiography, liver ultrasound and bone scintigraphy); patient age between 18 and 70 years; and provision by the patient of written informed consent to participation in the study and to central immunohistochemical examination of the tumour tissue.

Patients were excluded from participation if there was evidence of locally advanced (stage IIIB), bilateral, metastatic, or inflammatory breast cancer and if there had been previous treatment for breast cancer including surgery, radiotherapy or cytotoxic or endocrine treatments (surgical diagnostic procedures were allowed). Participating centres had to confirm that the trial was conducted according to the protocol recommendations, and had to apply for approval from an ethics committee. The study was conducted in accordance with the Helsinki Declaration. Complete source-data verification was provided by bi-monthly visits from an external clinical research organisation. The entry of data into the central database was double-checked and cross-checked by the responsible data-verifiers and data-managers.

### Study treatment and assessments

All patients received doxorubicin at a dosage of 50 mg/m^2 ^and docetaxel at a dosage of 75 mg/m^2 ^every 14 days for four cycles (ddAT). If they were randomly assigned to receive chemoendocrine treatment, patients received tamoxifen as a 30-mg tablet once daily in the morning, beginning on day 1 of the first cycle. All patients received lenograstim or filgrastim subcutaneously on days 5 to 10. After completion of chemotherapy and assessment of the response, all patients underwent surgery. Surgery had be performed 14 to 28 days after the last chemotherapy cycle, which was 8 to 10 weeks after the initiation of systemic therapy.

The size of the breast lump and axillary nodal status was determined by palpation before each cycle and before surgery. The product of the two largest perpendicular diameters was used to approximate the tumour area. In patients with multifocal or multicentric breast cancer, the lesion with the largest diameter was chosen for follow-up.

Clinical response after two cycles was defined according to the following criteria: complete response (CR) when no breast tumour was palpable; partial response (PR) when the reduction in the tumour area was ≥ 50%; and no change (NC) when the tumour area was reduced < 50% or increased < 25%. Progressive disease (PD) was recorded if the tumour area increased ≥ 25%, or if a new lesion was detected. In the surgical specimen, the classification of histological response proposed by Sinn *et al*. [22] was used (grade 0: no effect; grade I: resorption and tumour sclerosis; grade II: minimal focal invasive residues of ≤ 5 mm; grade III: only non-invasive tumour residues; grade IV: no viable tumour cell detectable). Only grade IV regression was considered to represent as a pathological complete response.

### Histopathological and immunohistological studies

The core-cut specimens from the study patients were fixed in 4% (w/v) phosphate buffered formalin and embedded in paraffin at the participating site, and then collected centrally for further examination. A haematoxylin/eosin-stained section of each block was prepared for central confirmation of the histological diagnosis and determination of the histological type and grade [23]. Serial sections of 2 to 3 μm were mounted on capillary-gap slides (DAKO Diagnostica, Hamburg, Germany) and dried at 37°C. Immunohistochemical staining was performed within 1 week. Slides were incubated at 60°C for 60 min and deparaffinised (2 × 5 min xylene followed by 5 min each ethanol 95%, 90%, 70% and 2 × 1 min distilled water), and treated with antigen retrieval buffer (DAKO) in a microwave oven to unmask the antigens. Automated capillary-gap technology staining with DAKO Techmate was carried out to provide identical staining conditions. The following antibodies (Ab) were used: ER: 1D5 (DAKO), dilution 1:100; PgR: polyclonal Ab (DAKO), dilution 1:100; Ki-67: MIB1 (Dianova, Hamburg, Germany) dilution 1:200; HER2: polyclonal Ab A0435 (DAKO), dilution 1:2,000; p53: mouse monoclonal antibody DO7 (DAKO), dilution 1:100, bcl-2: 124 (DAKO), dilution 1:100. Tissue sections were incubated with the primary antibodies for 25 min. As secondary antibodies, we used a DAKO kit for 25 min. Endogenous peroxidase was blocked with kit supplied H_2_O_2_. AEC (DAKO) was used as a chromogen substrate, and slides were slightly counterstained with haematoxylin.

Immunostaining was semiquantitatively graded according to the proportion of positive cells. Tumours were considered ER-positive or PgR-positive when 10 to 100% of all tumour cells had nuclear staining for oestrogen receptor or progesterone receptor, respectively. Tumours were categorised into three groups in relation to the proliferative activity: low (0 to 15% of tumour cells with nuclear staining for Ki-67), medium (16 to 30%), and high (31 to 100%). Normal p53 expression was recorded when 1 to 50% of the tumour cells were positively stained. Abnormal expression was detected when no tumour cells, or 51 to 100% of the tumour cells, were positively stained. HER2 staining was scored on a scale of 0 to 3+ using the scoring system outlined in the DAKO Hercept Test. Only unambiguous membrane staining was evaluated. Only HER2 3+ tumours were regarded as positive for overexpression. bcl-2 was scored semiquantitatively (0+ to 3+) judging the cytoplasmatic expression of bcl-2, but tumours with 2+ and 3+ staining were regarded as having normal expression. All histological evaluations were carried out by two independent investigators (HPS, GvM).

### Statistical evaluation

The probability of a pCR was estimated using (1) a univariate logistic regression model for the following clinical factors recorded at randomisation: menopausal status, tumour size, nodal status, grade, and clinical response after two cycles of ddAT; and (2) by immunohistochemistry findings: oestrogen and progesterone hormone receptor status, Ki-67, HER2, p53, and bcl-2. In a multivariate logistic regression analysis, immunohistochemical markers alone, and clinical and biological markers together and separated by treatment group, were evaluated. Here, oestrogen and progesterone receptor status were combined to make the 'hormonal receptor' factor, which is positive when at least one of the underlying factors is positive. All factors were included in a backward logistic regression model. Patients with missing values were excluded from the corresponding analysis. p Values greater than 0.05 were reported as not significant (NS). In addition, a multivariate logistic regression model for clinical response after two cycles was established in order to assess the predictive value of baseline factors on this intermediate measure.

## Results

A total of 250 patients were recruited into the trial between April 1998 and June 1999 by 56 participating centres all over Germany. A total of 973 of 996 (97.7%) planned cycles of ddAT were administered. The overall pCR rate for all patients was 9.7%, as previously reported [21]. Sufficient tumour tissue and information about pCR was detectable in 196 tumour samples. The availability of core-cut biopsies and the detection of biomarkers are listed in Table 1.

**Table 1:** Progress of tumour biopsies throughout the study

	**Patients (n)**
Patients randomised	250
To ddAT + tamoxifen	122
To ddAT – tamoxifen	128
Data on pCR available	247
No breast cancer	1
Chemotherapy refused	1
Surgery refused	1
Core biopsies available and evaluable	196
Not available	35
No tissue on block	6
Non-characteristic tissue	2
No tumour tissue on block	8
Results available for multivariate analysis (without clinical response after 2 cycles)	193
Multivariate analysis data missing for:	
Menopausal stage	0
Tumour size	0
Nodal status	0
Grade	2
ER	0
PgR	0
Ki-67	0
HER2	2
p53	0
bcl-2	0
Results available for multivariate analysis (with clinical response after 2 cycles)	181
Multivariate analysis data missing for clinical response after two cycles	12

ddAT, dose-dense Adriblastin (Doxorubicin) and Taxotere (Docetaxel); ER, oestrogen receptor; HER2, human epidermal growth factor receptor 2; pCR, pathological complete remission; PgR, progesterone receptor.

Patients were premenopausal in 54.1% of the 196 analysed cases. The tumours had a palpable diameter of more than 4 cm in 82 patients (41.8%). In all, 98 patients (50.0%) did not have palpable enlarged axillary lymph nodes. The histological differentiation was centrally determined as grade 1 in six tumours (3.1%), as grade 2 in 84 tumours (43.3%) and as grade 3 in 104 tumours (53.6%). A total of 96 patients (51.9%) had a clinical complete or partial response after two cycles of ddAT. ER-positive and PgR-positive tumours were found in 56.1% and 39.3%, respectively. In all, 46 tumours (23.7%) showed a HER2 overexpression with a score of 3+. Proliferation activity of the tumours was low in 38.3% and high in 39.8%. p53 and bcl-2 were normally expressed in 91 (46.4%) and 72 (36.7%) patients, respectively. All characteristics were found to be well balanced in the two treatment arms (Table 2).

**Table 2:** Distribution of predictive factors in relation to subgroups and in relation to treatment, with corresponding rates for pCR

	**ddAT – Tamoxifen (n = 98)**			**ddAT + Tamoxifen (n = 98)**		
	
	**n**	**%**	**pCR (%)**	**n**	**%**	**pCR (%)**
Menopausal status:						
Premenopausal	50	51.0	10.0	56	57.1	12.5
Peri-/postmenopausal	48	49.0	8.3	42	42.9	2.4
Tumour size:						
≤ 4 cm	58	59.2	12.1	56	57.1	8.9
> 4 cm	40	40.8	5.0	42	42.9	7.1
Clinical nodal status:						
Negative	52	53.1	15.4	46	46.9	13.0
Positive	46	46.9	2.2	52	53.1	3.9
Grade:						
I/II	46	47.9	4.3	44	44.9	0
III	50	52.1	14.0	54	55.1	14.8
Clinical response after two cycles:						
cPR/cCR	45	48.9	13.3	50	54.3	12.0
cNC/cPD	47	51.1	4.3	42	45.7	4.8
ER:						
0 to 9%	40	40.8	17.5	46	46.9	17.4
10 to 100%	58	59.2	3.5	52	53.1	0
PgR:						
0 to 9%	55	56.1	14.5	64	65.3	12.5
10 to 100%	43	43.9	2.3	34	34.7	0
Ki-67:						
0 to 15%	42	42.9	2.4	33	33.7	6.1
16 to 30%	21	21.4	0	22	22.5	4.5
31 to 100%	35	35.7	22.9	43	43.9	11.6
HER2:						
0 to 2+	73	76.0	9.6	75	76.5	10.7
3+	23	24.0	8.7	23	23.5	0
p53:						
1 to 50%	47	48.0	6.4	44	44.9	4.5
0 + 51 to 100%	51	52.0	11.8	54	55.1	11.1
bcl-2:						
0 to 1+	60	61.2	13.3	64	65.3	12.5
2 to 3+	38	38.8	2.6	34	34.7	0

cCR, clinical complete response; cNC, clinical no change; cPD, clinical progressive disease; cPR, clinical partial response; ddAT, dose-dense Adriblastin (Doxorubicin) and Taxotere (Docetaxel); ER, oestrogen receptor; HER2, human epidermal growth factor receptor 2; pCR, pathological complete remission; PgR, progesterone receptor.

The pCR rates in the various subgroups in relation to the various clinical and biological factors are shown in Table 3. A pCR rate below 4% was found in patients with ER-positive or PgR-positive tumours, normal bcl-2 status, low and medium proliferation activity, grade I/II differentiation, no clinical response after two cycles of chemotherapy, or positive clinical nodal status. A probability of a pCR of more than 15% was found in patients with ER-negative tumours or high proliferation activity.

**Table 3:** Univariate logistic regression for the predicting of a pCR irrespective of study treatment

	**pCR**		**Odds ratio**	**95% CI**
			
	**n**	**%**		
Menopausal status:				
Premenopausal	12	11.3	2.17	0.73 to 6.41
Peri-/postmenopausal	5	5.6		
Clinical tumour size:				
≤ 4 cm	12	10.5	1.81	0.61 to 5.36
> 4 cm	5	6.1		
Clinical nodal status:				
Negative	14	14.3	5.28	1.47 to 19.00
Positive	3	3.1		
Grading:				
Grade I + II	2	2.2	0.13	0.03 to 0.61
Grade III	15	14.4		
Clinical response after: 2 cycles ddAT				
cCR/cPR	12	12.6	3.07	0.95 to 9.91
cNC/cPD	4	4.5		
ER:				
0 to 9%	15	17.4	11.41	2.53 to 51.41
10 to 100%	2	1.8		
PgR:				
0 to 9%	16	13.5	11.81	1.53 to 90.97
10 to 100%	1	1.3		
Ki-67:				
0 to 15%	3	4.0	0.32	0.09 to 1.15
16 to 100%	14	11.6		
HER2:				
0 to 2+	15	10.1	2.48	0.55 to 11.28
3+	2	4.3		
p53:				
1 to 50%	5	5.5	0.45	0.15 to 1.33
0 + 51 to 100%	12	11.4		
bcl-2:				
0 to 1+	16	12.9	10.52	1.36 to 81.09
2 to 3+	1	1.4		

cCR, clinical complete response; cNC, clinical no change; cPD, clinical progressive disease; cPR, clinical partial response; ER, oestrogen receptor; HER2 human epidermal growth factor receptor 2; pCR, pathological complete remission; PgR, progesterone receptor.

The clearest differences between the pCR rates for the two treatment groups were seen in relation to Ki-67 and HER2. Patients with a low Ki-67 percentage showed a better response when treated with tamoxifen, whereas patients with a high Ki-67 percentage did not benefit from tamoxifen treatment. HER2-overexpressing tumours showed a similar response to that in HER2-negative patients who were treated without tamoxifen, but when patients with HER2-positive tumours were treated with ddAT plus tamoxifen, no pCR was detected. Highly differentiated ER-positive or PgR-positive tumours were never completely eradicated with chemoendocrine treatment (Table 2).

In univariate logistic regression models, negative lymph nodes, poor tumour differentiation, negative ER, negative PgR and loss of bcl-2 were found to be significantly predictive of a pCR. The highest odds ratios were found for ER, PgR and bcl-2, with a more than 10 times higher chance of a pCR when the expression changed from favourable to unfavourable (Table 3).

When only the experimental biological factors were included in a multivariate logistic regression model, bcl-2 was found to be a significant predictor of the efficacy of systemic therapy. Patients with low bcl-2-expressing tumours achieved a pCR 9.4 (1.17 to 75.18) times more often than those who had tumours with normal bcl-2 levels (Table 4).

**Table 4:** Biomarkers in relation to their predictive value for achieving a pathological complete response: multivariate logistic regression in 194 patients

	**Odds ratio**	**95% CI**	**p Value**
Ki-67	0.43	0.11 to 1.61	0.208
HER2	4.05	0.87 to 18.95	0.076
p53	0.69	0.22 to 2.16	0.527
bcl-2	9.39	1.17 to 75.18	0.035

HER2, human epidermal growth factor receptor 2.

For more complex models, oestrogen and progesterone receptor status were combined to form the factor 'hormonal receptor', which was defined to be positive if at least one of the receptors, oestrogen or progesterone, is positive. If both receptors are negative, hormonal receptor status is also negative. When all factors, established and experimental, were included in the multivariate logistic regression model, clinical nodal status and hormonal receptor were found to be significant predictors of pCR. When clinical response after two cycles was excluded from the analysis, the same factors were still significant predictors (Table 5). Using the backward stepwise elimination procedure (significance level 10%), a clinical response after two cycles, lymph-node status, and hormonal receptor were found to be significant predictors of a pCR. If a clinical complete or partial response occurred after two cycles, patients had a 3.3 times higher chance of achieving a pCR at surgery than patients without a response. Patients with hormonal receptor negative tumours had a 24.3 times higher probability of achieving a pCR than patients with PgR-positive tumours. A patient with clinically uninvolved axillary lymph nodes had a 5.0 times higher chance of a pCR than a patient with suspicious lymph nodes.

**Table 5:** Multivariate logistic regression analysis of established and experimental factors for predicting a pathological complete response, irrespective of study treatment: clinical response after two cycles excluded from second analysis

	**First analysis**			**Second analysis**		
	
	**Odds ratio**	**95% CI**	**p Value**	**Odds ratio**	**95% CI**	**p Value**
Menopausal status	1.63	0.44 to 6.05	0.467	1.89	0.55 to 6.51	0.312
Tumour size	1.75	0.46 to 6.68	0.411	1.80	0.51 to 6.40	0.363
Clinical nodal status	5.48	1.24 to 24.16	0.025	5.44	1.35 to 21.87	0.017
Grade	0.21	0.03 to 1.33	0.098	0.16	0.03 to 0.91	0.039
Clinical response after two cycles	3.68	0.90 to 15.03	0.069	-	-	-
Hormonal receptor	27.00	2.21 to 330.57	0.010	7.83	1.24 to 49.42	0.029
Ki-67	0.75	0.16 to 3.56	0.716	0.78	0.18 to 3.39	0.740
HER2	4.09	0.70 to 24.08	0.119	3.66	0.69 to 19.30	0.126
p53	4.11	0.73 to 23.01	0.108	2.55	0.56 to 11.66	0.227
bcl-2	3.75	0.35 to 39.54	0.272	4.98	0.53 to 46.95	0.161

HER2, human epidermal growth factor receptor 2.

In addition, a multivariate regression model was performed to predict the intermediate variable clinical response after two cycles. Out of established and experimental factors, only bcl-2 proved to have a statistically significant impact on the clinical response after two cycles (Table 6).

**Table 6:** Multivariate logistic regression analysis of established and experimental factors for predicting a clinical response after two cycles, irrespective of study treatment

	**Odds ratio**	**95% CI**	**p Value**
Menopausal status	1.35	0.72 to 2.55	0.351
Tumour size	0.93	0.49 to 1.75	0.821
Clinical nodal status	1.04	0.56 to 1.94	0.903
Grade	0.67	0.33 to 1.38	0.279
ER	1.09	0.43 to 2.77	0.850
PgR	0.56	0.23 to 1.35	0.195
Ki-67	0.58	0.29 to 1.15	0.120
HER2	0.99	0.46 to 2.16	0.990
p53	1.43	0.69 to 2.96	0.338
bcl-2	2.75	1.27 to 5.97	0.011

ER, oestrogen receptor; HER2, human epidermal growth factor receptor 2; PgR, progesterone receptor.

The distribution of pCR rates across subgroups of patients is depicted in Table 7. It demonstrates that there is no significant change in the pCR rates in the treatment arms within these subgroups.

**Table 7:** Distribution of patients in subgroups by treatment with corresponding pCR rates

		**n (pCR)**	
		
		**ddAT – Tamoxifen (n = 98)**	**ddAT + Tamoxifen (n = 98)**
ER+	HER2+	11 (1)	10 (0)
	HER2-	46 (1)	42 (0)
	HER2 status missing	1 (0)	0 (0)
	Total	58 (2)	52 (0)
ER-	HER2+	12 (1)	13 (0)
	HER2-	28 (6)	33 (8)
	Total	40 (7)	46 (8)
Total		98 (9)	98 (8)

ddAT, dose-dense Adriblastin (Doxorubicin) and Taxotere (Docetaxel); ER, oestrogen receptor; HER2, human epidermal growth factor receptor 2; pCR, pathological complete remission.

## Discussion

Separating patients into groups depending on their predicted tumour responses may offer a significant clinical advantage in their management. The response to preoperative chemotherapy correlates significantly with disease-free and overall survival. The occurrence of a clinical complete response subsequent to the administration of neo-adjuvant chemotherapy may be associated with only partial eradication of occult metastatic disease. A pathological complete response is therefore considered to be the optimal criterion, as it correlates with a 5-year disease-free survival of 84% in stage I-IIIA disease, whereas a clinical complete response leads to a 5-year disease-free survival of only 76% [1,3,24,25]. Pathological complete response has approximately the same prognostic power as pathological lymph-node status [3], but whereas lymph-node status is an existing condition that can only be changed by early detection, the pCR rate can be improved by using better systemic treatments. The fact that long-term disease-free survival is not achieved in all patients suggests that disseminated tumour cells are selectively more resistant than the primary tumour. This has indeed been observed for lymph-node metastases, which are less responsive to preoperative systemic treatment than the primary tumour; the cause can probably be attributed to distant 'micrometastases'.

However, pCR as an intermediate end point can be achieved within weeks after the start of treatment, and is therefore an ideal way of comparing new active drugs. If achieving a pCR could be predicted even earlier, breast cancer patients could be saved from having to undergo ineffective treatment regimens that do not translate into a tumour response in the preoperative setting.

A variety of proven and putative predictive markers (for example, ER, PgR, ploidy, S-phase, HER2, p53, and other oncogenes and growth factors) have been evaluated in previous studies [19] in material obtained from fine-needle biopsies and core-cut biopsies, and have been correlated with the tumour response in order to assess whether unnecessary surgical or radiological treatment can be avoided after systemic therapy, or even whether systemic treatment can be avoided entirely. In addition, there have been studies in which serial biopsies were taken to examine changes in biological markers during therapy and correlate these changes with the treatment outcome. However, the populations examined were mostly below the critical number of 100 patients, and only preliminary and contradictory data were obtained.

As shown in Table 6, the predictive values of the factors studied are in fact strongly influenced by the type of treatment given. The combination of tamoxifen with chemotherapy is detrimental to the investigation of predictive markers, since more aggressive tumours react differently to chemotherapy, at least during the short term of an 8-week treatment of the type used in the present study, as proliferation is blocked by tamoxifen.

Admittedly, 196 patients are not sufficient to analyse a large number of predictive factors simultaneously, so only large differences can be distinguished. Another aspect that may be criticised is that immunohistochemistry is not the best method of detecting HER2 overexpression or p53 mutation, and this may be the reason why more significant results were not achieved. Moreover, the detection of a clinical response by palpation after the second cycle is observer-dependent, and might be replaced by more valid measurement methods such as breast ultrasonography or magnetic resonance imaging. However, all of these methods are currently widely used in clinical routine.

The response after two cycles is influenced by a wide variety of biological features representing the resistance or sensitivity of the tumour to a specific treatment. This is evident from Table 5, where it can be seen the impact of most factors disappeared when this response factor was added to the multivariate analysis. Since the response to chemotherapy has always been regarded as multifactorial, this factor seems more appropriate than using a single factor for predicting pCR.

One study of 198 patients with inflammatory breast cancer confirmed that the response after two cycles, together with tumour size and age, was a significant predictor of pCR [26]. In fact, in this study the response after two cycles was the only independent predictor in the multivariate analysis.

Recently, a French group has demonstrated that patients, who did not show a clinical response after three cycles of preoperative chemotherapy with vinblastine, thiotepa, methotrexate, and fluorouracil, achieved a secondary clinical response in 40% of cases with a salvage regimen including cisplatin, etoposide, fluorouracil, and mitomycin. Patients with a clinical response to the first regimen had a 5-year overall survival rate of 82%, compared with 67% in those who did not respond. However, in patients who responded to the second regimen, the prognosis improved up to 82%, comparable to that of initial responders, whereas patients with no response at all showed a poor outcome with a 57% survival rate [27]. In another pilot trial, 133 patients with large (> 3 cm) or locally advanced tumours and a clinical response to four cycles of cyclophosphamide, vincristine, doxorubicin and prednisolone (CVAP) were randomly assigned either to continue for a further four cycles with CVAP or to four cycles of docetaxel. The pathological complete response rate in patients with eight cycles of CVAP was 16%, compared with 34% in those with CVAP and docetaxel. The response rate of non-responders after salvage treatment with docetaxel was only 2% [28].

Both of these trials demonstrate that the response to chemotherapy after a small number of cycles is not only predictive of pCR and overall survival, but can also be influenced by non-cross-resistant treatments. As demonstrated in the presented study, the decision can already been taken after two cycles of therapy, and patients can be spared from ineffective therapy at a very early stage.

**Figure 1:**
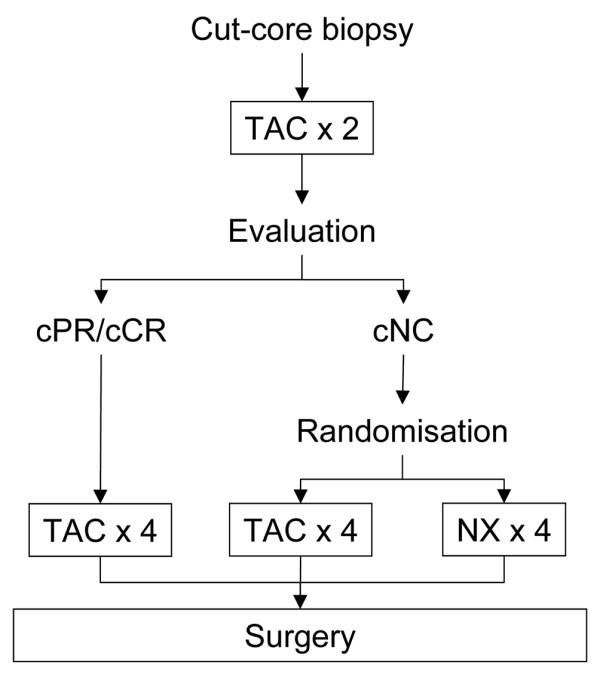
Schematic diagram of the study design. Proposed trial design for preoperative chemotherapy in operable breast cancer, exploring *in vivo *chemosensitivity as a decision-maker for the use of non-cross-resistant salvage chemotherapy in non-responding patients. TAC: Taxotere (docetaxel), Adriblastin (doxorubicin) and cyclophosphamide; NX: Navelbine (vinorelbine) and Xeloda (capecitabine).

The validity of this observation still needs to be confirmed in a large prospective randomised trial before the approach can be introduced into routine clinical practice. Our group has therefore initiated a phase III trial in which non-responders to two cycles of docetaxel, doxorubicin, and cyclophosphamide are being randomly assigned to either continuation for a further four cycles or to four cycles of a non-cross-resistant regimen consisting of vinorelbine and capecitabine (Figure 1) [29].

## Conclusion

Reliable prediction of a pathological complete response after preoperative chemotherapy is not possible with clinical and biological factors routinely determined before start of treatment. The response after two cycles of chemotherapy is a strong but dependent predictor. The only independent factor in this subset of patients was bcl-2.

## Abbreviations

cCR = clinical complete response; CI = confidence interval; cNC = clinical no change; cPD = clinical progressive disease; cPR = clinical partial response; ddAT = dose-dense Adriblastin (Doxorubicin) and Taxotere (Docetaxel); ER = oestrogen receptor; MRI = magnetic resonance imaging; pCR = pathological complete remission; PgR = progesterone receptor.

## Competing interests

The authors declare that they have no competing interests.

## Authors' contributions

GvM was responsible for the study design, data collection and participated in drafting the manuscript. H-PS was responsible for the study design, data collection, data evaluation and participated in drafting the manuscript. GR was responsible for study design and data collection. SL was responsible for data collection, analysis and drafting the manuscript. J-UB was responsible for study design and data collection. HE was responsible for study design and data collection. JH was responsible for study design and data collection. EM was responsible for study design and data collection. CJ was responsible for study design and data collection. SDC was responsible for study design and data collection. AC was responsible for study design, data collection, biometrical analysis and drafting the manuscript. MK was responsible for study design and data collection.
